# Revealing the sequence of interactions of PuroA peptide with *Candida albicans* cells by live-cell imaging

**DOI:** 10.1038/srep43542

**Published:** 2017-03-02

**Authors:** Nadin Shagaghi, Mrinal Bhave, Enzo A. Palombo, Andrew H. A. Clayton

**Affiliations:** 1 Department of Chemistry and Biotechnology, Faculty of Science, Engineering and Technology, Swinburne University of Technology, PO Box 218, Hawthorn, VIC, Australia; 2 Centre for Micro-Photonics, Faculty of Science, Engineering and Technology, Swinburne University of Technology, PO Box 218, Hawthorn, VIC, Australia

## Abstract

To determine the mechanism(s) of action of antimicrobial peptides (AMPs) it is desirable to provide details of their interaction kinetics with cellular, sub-cellular and molecular targets. The synthetic peptide, PuroA, displays potent antimicrobial activities which have been attributed to peptide-induced membrane destabilization, or intracellular mechanisms of action (DNA-binding) or both. We used time-lapse fluorescence microscopy and fluorescence lifetime imaging microscopy (FLIM) to directly monitor the localization and interaction kinetics of a FITC- PuroA peptide on single *Candida albicans* cells in real time. Our results reveal the sequence of events leading to cell death. Within 1 minute, FITC-PuroA was observed to interact with SYTO-labelled nucleic acids, resulting in a noticeable quenching in the fluorescence lifetime of the peptide label at the nucleus of yeast cells, and cell-cycle arrest. A propidium iodide (PI) influx assay confirmed that peptide translocation itself did not disrupt the cell membrane integrity; however, PI entry occurred 25–45 minutes later, which correlated with an increase in fractional fluorescence of pores and an overall loss of cell size. Our results clarify that membrane disruption appears to be the mechanism by which the *C. albicans* cells are killed and this occurs after FITC-PuroA translocation and binding to intracellular targets.

Antimicrobial peptides (AMPs) are natural defence molecules produced by the majority of living organisms, from microorganisms to humans. AMPs are excellent candidates to fight antimicrobial drug resistance. Compared to commercial antibiotics, it is a mystery how AMPs have been used successfully by nature for millions of years and remained so efficient during evolution^1^. AMPs often exert microbicidal effects, resulting from irreversible disruption of vital cellular structures and/or functions. The mechanism by which antimicrobial peptides act is a complex issue. It is essential to understand how these peptides act to entirely exploit the use of them as antimicrobial agents.

Many studies aimed to understand their mode of action have shown that AMPs attach to and insert into membrane bilayers to form stable transmembrane pores such as barrel-stave pores^2^ or toroidal pores^3^ or micellization in a detergent-like way (carpet mechanism)^4^. At the cell membrane, a critical threshold concentration needs to be reached to trigger a significant membrane disturbance. It proposed that the membrane-bound concentrations of peptides with minimum inhibitory concentration (MIC) values in the micromolar range can reach millimolar local concentrations and trigger disruptive effects on membranes^5^.

However, recent studies suggested that their membrane-compromising activity is not the only mechanism of microbial killing and their mechanism of action is much more complex and diverse^6^. It has been believed that the killing mechanism of AMPs depend on the membrane composition, the peptide concentration, and the final peptide ⁄ lipid ratio. Alternatively, at low peptide ⁄ lipid ratios, AMPs can translocate across cell membranes, disturbing their structure in a transient, non-lethal manner, and then reach their intracellular target^7^. Many AMPS have intracellular targets as their main mechanism of action or complementary to membrane perturbation. AMPs can inhibit the growth of pathogens, or kill it, by diverse mechanisms, including binding to nucleic acids^8^,^9^, inhibiting the syntheses of nucleic acids, proteins^10^,^11^, or cell-wall^12^, inhibiting enzymatic activities^13^,^14^ and altering the cytoplasmic membrane septum formation^15^. Therefore, the modes of action of AMPs have been categorized into two types: membrane lysis and non-membrane lysis.

It has become apparent that some AMPs apply simultaneous and multiple, independent or cooperative actions that probably result in their generally quick and potent antimicrobial activities. Some AMPs have a single mode of action which is concentration-independent, like apidaecin peptide which showed no lytic activity at any concentration tested^16^. In contrast, some AMPs, like Arasin 1, used a dual mechanism of action which was concentration-dependent; at concentrations above the MIC, Arasin 1 lysed membranes in a detergent-like manner, while at concentrations below MIC, Arasin 1 penetrated membranes and bound to intracellular targets^17^,^18^. However, previous biophysical studies gave only partial explanation of how the actual killing of the microbes occurs. Knowledge of the sequence of the molecular interactions that are necessary for antimicrobial activity of AMPS is lacking. When AMPs have transmembrane pore-forming mechanisms combined with intracellular targets, it is believed that the AMPs form those pores to reach their intracellular targets^19^,^20^,^21^,^22^.

The synthetic peptide PuroA (FPVTWRWWKWWKG-NH_2_), based on the unique tryptophan-rich domain (TRD) of the wheat endosperm protein, puroindoline A, showed antimicrobial activity against a number of bacteria, fungi and/or yeasts^23^,^24^,^25^. Vogel’s group, who firstly reported the antibacterial activity of PuroA in 2003 and suggested that it is a functional domain of puroindoline A, showed that this peptide preferentially binds to negatively charged vesicles and induces calcein leakage from these vesicles, suggesting it acts mainly on bacterial membranes and uses a lytic mechanism of action to exert its antimicrobial activity^23^. Recently, the same group suggested that the antibacterial activity of PuroA is more complex than originally assumed as the peptide bound to deoxyribonucleic acid (DNA), significantly inhibited macromolecular synthesis at sub-inhibitory concentrations in *Escherichia coli* and it also did not disrupt cytoplasmic membrane integrity in *E. coli* cells. Therefore, they proposed that the interaction of puroindoline peptides with bacterial membranes is only an initial step, followed by translocation into the bacterial cell, where they mainly exert their antibacterial effect^26^. Subsequently, Alfred and colleagues showed that *Saccharomyces cerevisiae* cells treated with PuroA appeared intact with pore-like structures in the membranes and leakage of extracellular material. Furthermore, and in agreement with Vogel’s findings, the peptide was found to bind to DNA *in vitro* and selectively permeabilised negatively charged vesicles. Therefore, they suggested that PuroA exerts its antimicrobial effects by disrupting the integrity of the cell membrane, followed by intracellular mechanisms of activity^20^.

These previous studies raise the following questions: what are the temporal dynamics of interaction between the peptide and its dual targets? Does pore formation precede DNA interaction, or vice-versa? Is cell death triggered by one or the other interaction, or are both involved? Finding the precise mechanism of action of PuroA is very significant for considering its applications in food, health and agriculture contexts. Therefore, in this study, we tried to answer these questions and we used time-lapse fluorescence lifetime imaging microscopy (FLIM) and time-lapse fluorescence microscopy to directly observe the localization and interaction kinetics of a fluorescently-tagged PuroA peptide on single *Candida albicans* cells in real time. Our results disclosed the detailed sequence of events during attack of *C. albicans* cells by PuroA, involving translocation and intracellular interaction followed by membrane pore formation and membrane disruption at the same peptide concentration.

## Results

### The effect of PuroA peptide on *C. albicans* morphology using SEM

**Figure 1:**
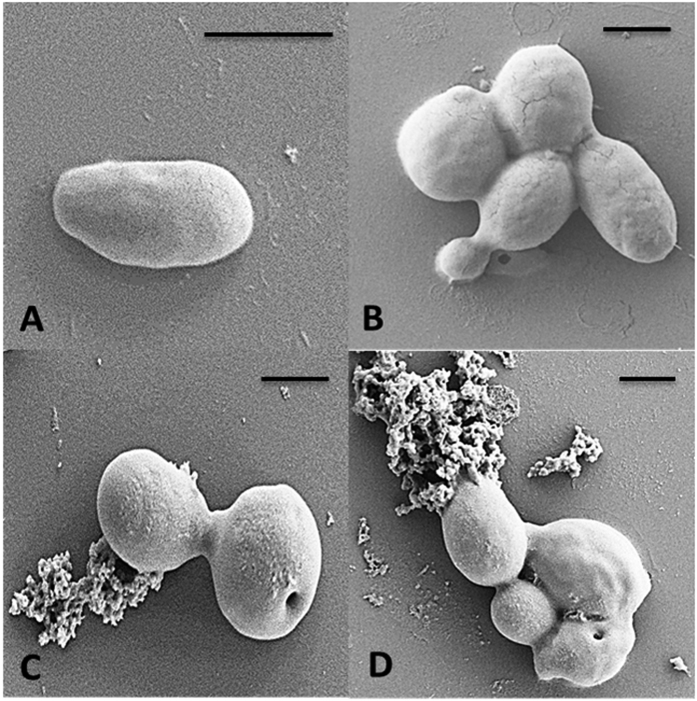
Scanning electronmicrographs of *C. albicans* treated with PuroA. (**A**,**B**) No-peptide control; (**C**). Puro A at 64 μg\mL; (**D**). PuroA at 125 μg\mL. Magnifications 20,000× , scale bar 2 μm. Pore size is 0.25–0.40 μm.

Control cells exposed only to PBS showed a regular cell shape with intact cell membrane (Fig. 1A and B). At 0.5 × MIC and 1 × MIC (64 μg/mL and 125 μg/mL, respectively), pores were observed on the membrane surface of some peptide-treated cells. Additionally, in some cells, there was extracellular material leaking from the cells (Fig. 1C and D).

### Time-kill assay

**Figure 2:**
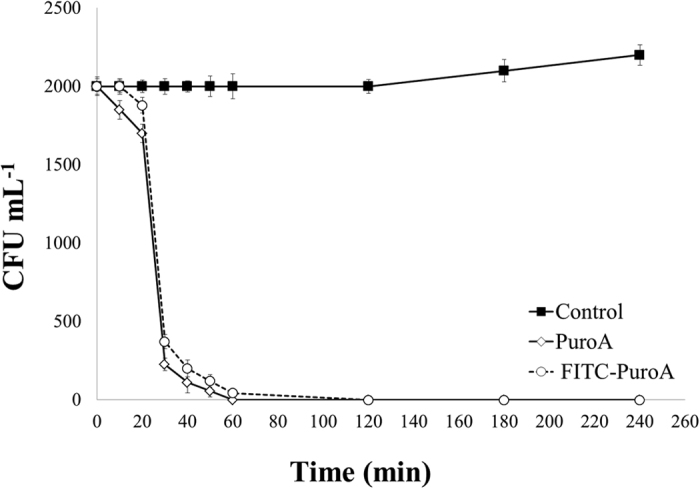
killing kinetics of PuroA and FITC-PuroA peptide. *C. albicans* cells, as indicated, were treated with peptides at MIC, and yeast survival was determined by viable cell counting at different times. Significant reduction of cell numbers was observed at 30 min. Data are presented as mean values ± standard deviations of three independent experiments.

The timescale to induce candidacidal effect on *C. albicans* cells by FITC-PuroA was determined by a time-kill assay. The peptide caused an 85% reduction in viable yeast cells at 30 min and complete killing (>99%) of all *C. albicans* cells at 60 mins. The time-kill kinetics were the same as with the unlabelled peptide (Fig. 2).

### Effect of PuroA on *C. Albicans* cell cycle

**Figure 3:**
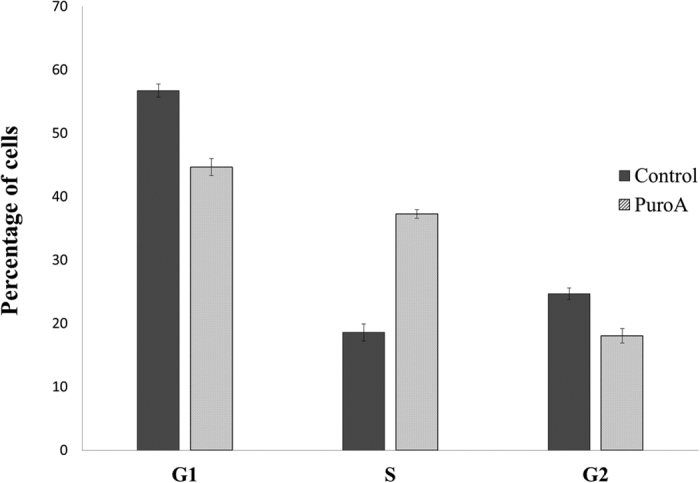
The effects of PuroA on the cell cycle progression of *C. albicans* cells. The cells were treated with PBS or 125 μg\mL of PuroA and their DNA contents was labelled with PI and analysed by flow cytometer. Histogram indicates the percentage of *C. albicans* cells in each phase of the cell cycle. Data are presented as mean values ± standard deviations of two independent experiments.

In order to investigate the effects of PuroA peptide on the intracellular physiology of *C. albicans* cells, particularly on DNA and/or protein synthesis *in vivo,* cell cycle analyses were performed for the yeast cells. The DNA content of *C. albicans* cells treated without/with 125 μg/mL of PuroA for 25 min was determined by flow cytometry after staining with PI which is known as DNA-intercalating agent. As shown in Fig. 3, in absence of PuroA, the percentages of cells in G1-phase, S-phases and G2-M phase were 56.73%, 18.58%, and 24.69%, respectively. After PuroA treatment, the proportion of *C. albicans* cells in the S-phase cell significantly increased to 37.28%, while that in G1-phase and G2-M phase decreased to 44.67% and 18.05%, respectively. Fluorescence intensity histogram profiles corresponding to Fig. 3 are shown in Fig. S1 (see Supplementary Information). The results suggest that PuroA arrested cell proliferation at the S-phase in *C. albicans,* resulting in inhibition of normal cellular processes.

### Time-lapse confocal microscopy

**Figure 4:**
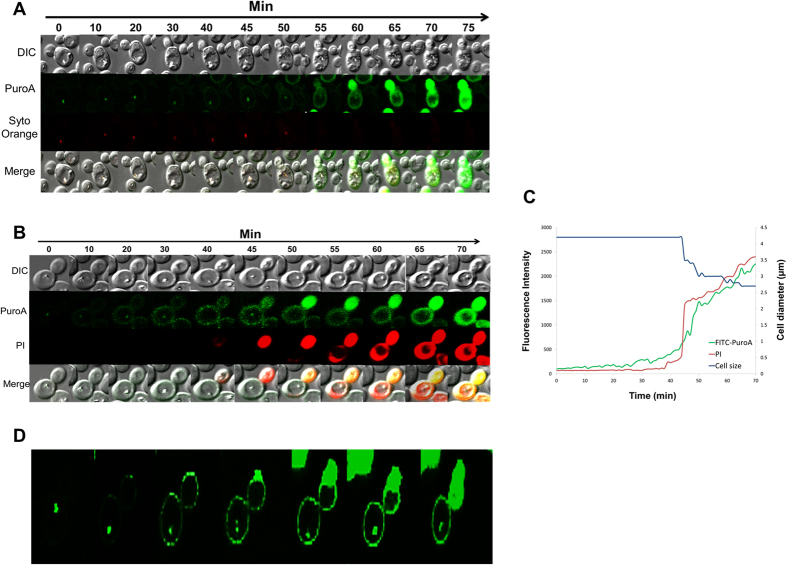
Sequence of events during attack of a single *C. albicans* cell by FITC-PuroA at 8 μg\mL, time-lapse confocal microscopy images taken in real time. (**A**) Cell nucleus stained with SYTO 85 Orange before addition of FITC-PuroA; the snapshots were taken from a single movie at the times shown at top. (**B**) PI was added simultaneously with the peptide, the snapshots were taken from a single movie at the times shown at top, right scale shows total intensity of green and red fluorescence associated with the cell vs. time over 70 min. (**C**) For the same cell in b, plots of total FITC intensity (green line), total PI intensity (red line) and cell size (blue line) vs time, only one single cell was amenable to quantitative analysis. (**D**) Single colour experiments using only FITC-PuroA.

We monitored the dynamic sequence of events in the antimicrobial action of PuroA peptide at 8 μg/mL using time-lapse confocal microscopy (Fig. 4). In single and/or two-colour experiments, PuroA was labelled with a green-fluorescent FITC probe to monitor the peptide localization in time. The green channel monitored emission from the labelled peptide and the red channel monitored emission from the DNA stain SYTO 85 Orange and PI; SYTO 85 Orange is a cell-permeant nucleic acid stain whereas PI is a cell-impermeant nucleic acid stain that enters the cell only when the membrane is comprised, binds to nucleic acids, and fluoresces strongly. Interleaved phase contrast microscopy observations allowed monitoring of changes in cell morphology with time.

To our surprise, we found that within 30 seconds of exposing the yeast cells to the peptide, FITC-puroA appeared to be located at the cell nucleus. This was confirmed via the strong colocalisation of the FITC-puroA fluorescence with the red fluorescence from the nucleic acid stain SYTO 85 Orange (Fig. 4A). The localization of the peptide in the nucleus persisted for about 20 minutes.

After 20–40 minutes (Fig. 4A and B), the peptide accumulated at the cell membrane, as evidenced by the increased cell surface fluorescence, and in some cells, enlargement of the cell’s vacuole was observed via phase contrast (Fig. 4B).

Forty minutes after peptide addition, peptide-induced disruption of membranes was observed by PI influx into the cell cytosol. The PI distribution was not uniform throughout the cell suggesting PI entry was localised (Fig. 4B). In parallel to the cytosolic influx of PI, a decrease in cell size was observed (by 35%) but complete dissolution of the cell was not apparent at this time point. These dynamic changes are summarized in Fig. 3C whereupon the average signals from peptide, PI and phase contrast from a single cell are plotted as a function of time.

### Time-lapse FLIM

To determine the interaction kinetics of peptide-peptide, peptide-membrane and peptide-nucleic acids interaction on the nanometer scale (i.e. <10 nm), we recorded fluorescence lifetime images as a function of time after adding FITC-PuroA peptide. The fluorescence lifetime of peptide changes when its state changes, i.e. peptide free in solution, peptide participating in pore formation, and bound peptide^27^,^28^.

**Figure 5:**
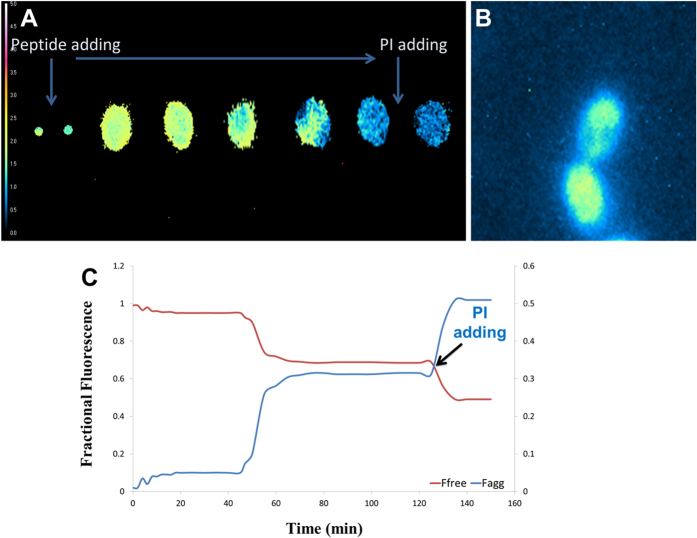
Time-lapse fluorescence lifetime images of FITC-PuroA interacting with a single *C. albicans* cell. (**A**) Fluorescence lifetime images of a single *C. albicans* cell before and after adding the peptide. Note the quenching in lifetime of the SYTO 85 Orange-stained nucleus after addition of the peptide. (**B**) Fluorescence intensity image of *C. albicans* cell during pore formation stage. (**C**) Fractional fluorescence from free PuroA, pore forming PuroA and nucleic acids bound PuroA, as a function of time Fluorescence intensity image of *C. albicans* cell during pore formation stage.

FITC-PuroA peptide bound to its intracellular target immediately after addition to yeast cell, as evidenced by the quenching in the FITC-PuroA fluorescence lifetime from 2.5 ns to 2 ns. In the experiments where the cells’ nuclei were stained with SYTO 85 Orange (Fig. 5a), a further reduction in the fluorescence lifetime of the FITC-PuroA peptide was observed (from 2 ns to 1.8 ns) indicating nanoscale proximity to the SYTO dye. After a lag time of about 35 to 45 mins, the pore formation stage started as detected from the decrease in the fluorescence lifetime of the FTC-PuroA at the cell membrane (lifetime dropped from 2 ns to 1.6–1.3 ns). This observation is supported by the finding that the aggregated form is substantially quenched, compared to the monomer form^29^ and also the fluorescence lifetime of fluorescent labelled AMPs was quenched upon pore formation^30^. In the context of a simple two-state pore/non-pore model, the calculated fractional fluorescence of pores increased from 7% during the lag phase, to 25–30% at 50 minutes and plateaued at 75% pore fraction at 60 minutes and beyond (Fig. 5c).

Addition of PI to the cell after pore formation and subsequent PI influx confirmed that the cell membrane was compromised. There was further quenching in fluorescence lifetime of the FITC-PuroA inside the cell when PI influx took place, again giving clear evidence of nanoscale interactions between FITC-PuroA and PI-nucleic acids in the cytosol.

## Discussion

**Figure 6:**
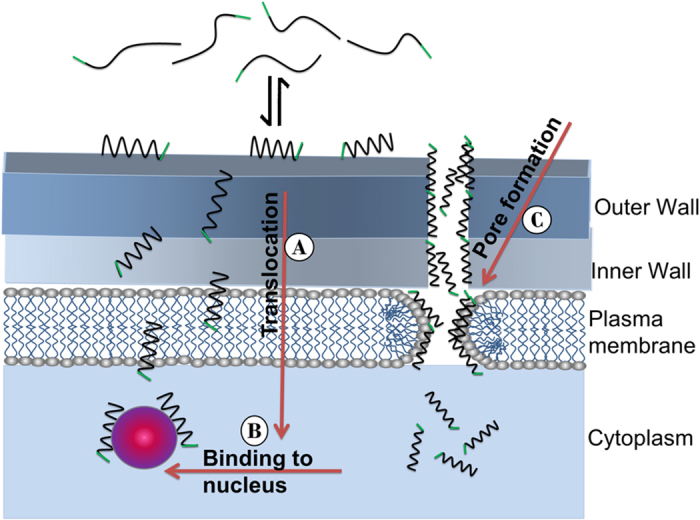
Schematic showing the sequence of the three events (**A**,**B** and **C**) occurring upon interaction of PuroA peptides with *C. albicans* cell.

The major findings in this work are how PuroA attacks *C. albicans* cells in real time and determining the main target that causes cell death. For most membranolytic AMPs, the cell membrane appears to be the primary target of these molecules and the intracellular targets generally considered the secondary targets after causing membrane damage. Somewhat surprisingly, we showed here that PuroA-FITC can translocate the cell membranes and reach their intracellular targets well before forming pores into membrane bilayers and disrupting the membrane integrity. FITC-PuroA showed three distinct phases of attack. The first phase involved translocation of FITC-PuroA across the cell membranes and binding to nucleic acids in the nucleus without permanent disruption of membranes. The second phase involved FITC-PuroA accumulation at the cell membrane and forming pores causing membrane disruption and PI and peptide influx and a decrease in cell size. In the third phase, peptide in the cytosol bound to nucleic acids (Fig. 6). These findings highlight the importance of assessing the interactions between AMPs and target cells using a range of exposure times to enable complete elucidation of the AMP’s mechanisms of action. These real time imaging experiments revealed three distinct events which occurred at different times at the same peptide concentration.

Clear pores were observed on the surface of the *C. albicans* cells incubated with PuroA for 1 h. Previously, PuroA showed *in vivo* and *in vitro* DNA binding ability^20^,^26^. Thus, the expected candidacidal mechanism of this peptide was formation of pores in the cell membrane, followed by intracellular mechanisms of activity. However, surprisingly, live cell imaging revealed that the peptide appeared inside the cell before the pore formation stage. Thus, pore formation is secondary to rather than concurrent with PuroA entry to cells. This emphasises the importance of live cell imaging in real time in understanding the mechanism of action of Puro A in this instance.

The distinct temporal dynamics of the peptide location and interaction may provide clues as to the precise mechanism of cell killing in this case. From the time-kill assay on a population of cells, it is clear that cell killing is not immediate but requires a lag phase of tens of minutes before a discernible drop in CFU is observed. Moreover, the significant reduction in the number of treated cells with PuroA after 45 min is consistent with the timescale of pore formation and loss in membrane integrity observed in our single cell assay. This would suggest that pore formation and disruption of the cellular integrity is the main mechanism of cell killing, and membrane permeabilization and cell shrinkage are effects from which the cells cannot recover. The loss in cell volume (cell shrinkage) in parallel to the permeabilization effect during attack by AMPs was also observed previously in *C. albicans* when treated with chicken cathelicidin-2 (CATH-2) peptide^31^ and in the Gram-positive bacterium *Bacillus subtilis* when treated with LL-37 and alamethicin^32^,^33^. On the intracellular level and before forming pores, this cationic peptide seemed to interact with the cell’s nucleic acids and inhibited some functions in the nucleus. From the flow cytometric analysis of the cell cycle and DNA distribution, it was determined that PuroA prevented cells from entering the G2 phase of the cell cycle, resulting in the accumulation of cells in S phase where DNA replication occurs. We stress that these observations apply only to *C. albicans,* and the relative importance of membrane disruption versus peptide-nucleic acid interactions on cell impairment may depend on the exact microorganism under examination.

*C. albicans* is an opportunistic pathogen that causes oral candidiasis and other conditions in immune-compromised individuals^34^. The *C. albicans* cell wall is mainly (80–90%) composed of carbohydrate to protect the cells from osmotic stress and maintain structural integrity^35^. In less than one minute, PuroA translocated the outer and inner cell-walls that are composed of polymers of mannose (mannan), mannoproteins^36^, polymers of glucose and polymers of N-acetyl-D-glucosamine (GlcNAc) containing β-1,4 bonds (chitin)^37^, as well as translocating the cytoplasmic membrane and binding to the nucleus. This rapid translocation through cell wall and cytoplasmic membrane and binding to nucleus could be due its unique tryptophan-rich domain^38^.

Other AMPs like the histidine-rich histatin 5 (Hst 5) peptide bound to laminarin (β-1,3-glucan) at the cell wall of *C. albicans* then translocated through cell well and cytoplasmic membrane in a non-disturbing manner. After translocation, uniform PI entrance occurred due to vacuolar expansion; PI uptake was a consequence of ionic efflux and vacuole expansion caused by cytosolic translocation of Hst 5^39^. However, previously, It proposed that the uptake of Hst-5 is a dichotomous event; at low concentrations, the peptide is internalized to the vacuole via receptor-mediated endocytosis, whereas at high conditions, Hst-5 cytoplasmic uptake occurs through a single break site on the plasma membrane^40^. On the other hand, our data clearly show that PI entry occurs well after translocation of PuroA through a single point in the cell membranes and is due to a substantial accumulation of PuroA at that point and forming pores with all these events happening at the same peptide concentration.

Our results reveal that live cell imaging approaches can be a useful adjunct to other approaches for investigating the mechanisms of action of antimicrobial peptides. First, FLIM provides a measure of the nanoscale interactions of peptides with other labelled molecules or cellular constituents in real time. Second, imaging provides a measurement of the localization of the peptide relative to membranes, cytosol, nucleus or other organelles. By combining these biophysical methods with biological assays (e.g. time-kill assay), the kinetics of peptide interactions and the kinetics of cell death can be compared leading to mechanistic insights.

It is important to stress that the results gleaned in the present paper are specific to the peptide and cells being investigated. Indeed, we might expect that the balance of intracellular and membrane disruption interactions to be cell-type specific and to also depend on other variables as well. The methods utilized here will be useful in investigating these issues in future work.

## Material and Methods

### Materials

C-terminal amidated PuroA (FPVTWRWWKWWKG-NH_2_) and FITC-labelled PuroA (FITC-FPVTWRWWKWWKG-NH_2_) were synthesized with >95% purity by solid-phase methods using N-(9-fluorenyl) methoxycarbonyl (Fmoc) chemistry at Biomatik Crop (Ontario, CA). Peptides used without further purification. Peptide solutions were made in sterile phosphate-buffered saline (PBS; 140 mM NaCl, 2.5 mM KCl, 1.6 mM KH_2_PO_4_, 15 mM Na_2_HPO_4_, pH 7.4). A 20 mM solution (in DMSO) of propidium iodide and A 5 mM solution (in DMSO) of SYTO 85 Orange fluorescent nucleic acid stain were purchased from Molecular Probes (L7012 and S11366, respectively). All aqueous solutions were made in sterile, ultrapure (18 MΩ) water which was quartz-distilled and deionised in a Milli-Q^TM^ system (Millipore, Bedford, MA, USA).

### Yeast Strains and Cell Cultures

*Candida albicans* (FRR 5580) was obtained from CSIRO Food Fungal Culture Collection. Yeast stock was prepared in 25% glycerol Potato Dextrose Broth (PDB; Difco-BD, USA) and stored at –80 °C until needed. Prior to each experiment, the yeast culture was refreshed from stocks on Potato Dextrose Agar (PDA), and a fresh yeast suspension was grown overnight at 30 °C with shaking (200 rpm) in PDB.

For live cell imaging, a yeast suspension (1 × 10^6^ cells) was deposited onto a chambered coverglass (Nunc™ Lab-Tek™ II, Thermo Scientific); the chamber was previously coated with 0.01% poly-L-lysine. The cells were allowed to adhere, and then any loosely-adhered cells were gently rinsed away with PBS solution. FITC-labelled PuroA was added to give a final concentration of 8 μg/mL; the peptide was used at this concentration to delay the antimicrobial process and the interaction kinetics, so they could be measured in a timescale of minutes. The peptide solution was made immediately before the experiment and was vortexed (3,200 rpm) for at least 30 s. In some experiments, the nuclei of yeast cells were stained with SYTO 85 Orange prior deposit onto the chamber.

### Fluorescence lifetime imaging microscopy (FLIM)

All FLIM experiments were performed using a LIFA instrument (Lambert Instruments, Leutingwolde, The Netherlands) attached to an inverted microscope (Ti Eclipse, Nikon Inc., Japan). The samples were observed through a 100 X NA 1.2 oil objective (Nikon Plan-Fluor, Nikon Inc, Japan). The fluorescence excitation source was a 474 nm LED with a sinusoidal modulation frequency of 35 MHz (Lambert Instruments, Leutingwolde, The Netherlands). The emission was passed through an acousto-optic tunable filter (Gooch and Housego) and imaged onto an image intensifier (Lambert Instruments, Leutingwolde, The Netherlands). Phase and modulation lifetimes were determined by taking a series of 12 phase images of differing phase shift (fluorescence lifetime image stack), utilizing the LI-FLIM software package (Version 1.2.3.11) supporting the LIFA instrument. Fluorescence lifetime image stacks were recorded after adding the peptide for up to 4 hours (typically 1–4 minute intervals). In some experiments, propidium iodide (Molecular Probes^®^, USA) was added to the yeast/peptide solution to give a final concentration of 5 μg/ml. Rhodamine 6 G was used as a reference (lifetime R6G: 4.1 ns). Fluorescence lifetime image stacks were recorded at 520 nm, 580 nm and 620 nm.

Peptide-nucleic acid interactions in the nucleus of intact single yeast cells were measured by the quenching of the fluorescein lifetime from the FITC-PuroA peptide via fluorescence resonance energy transfer to a nucleic-acid bound SYTO 85 Orange acceptor. The lifetime of the FITC-PuroA fluorescence bound to the nucleus was measured and then compared to the lifetime of the FITC-PuroA bound to the nucleus of a cell counter-stained with SYTO 85 Orange.

Peptide-nucleic acid interactions in the cytosol of membrane-compromised single yeast cells were measured by quenching of the FITC-PuroA peptide lifetime upon addition of propidium iodide (PI). The lifetime of the FITC-PuroA peptide was measured without PI and then measured after addition of PI.

Peptide-peptide interactions were measured through lifetime self-quenching of the fluorescein dye on FITC-PuroA.

The FLIM data were converted to phasor space, where x = mcosQ and y = msinQ, where m is the modulation and Q is the phase^41^,^42^,^43^. Then the fluorescence was decomposed into fractional states, the fractional fluorescence from free peptide and peptide pore states (aggregate) were calculated. For a given phasor, r(x, y), the fractional fluorescence from the peptide pore state, f_pore_, is given by:


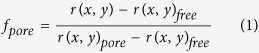



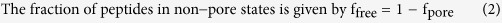


And the ratio of peptide concentration in the free and pore states is given by


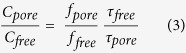


### Confocal laser-scanning microscopy

Confocal images were acquired with an inverted confocal microscope (FluoView FV1000; Olympus, AU), using a 100 X oil immersion objective. A 488-nm helium neon laser was used for excitation of FITC-labelled PuroA and a 561-nm helium neon laser was used for excitation of both PI and SYTO 85 Orange. The imaging sequence cycled among 488 nm fluorescence excitation, 561 nm fluorescence excitation, and differential interference contrast (DIC) with each sequence repeated every 30 s. All images were acquired and analyzed with Olympus FluoViewer software. ImageJ software (version 1.50b) was used for measuring the cell size.

### Scanning Electron Microscopy (SEM)

SEM was used to examine the effect of the peptides on the morphology of vegetative *C. albicans* cells. Cultures of *C. albicans* were cultivated overnight at 30 °C in PDB, and then made to 1.5 × 10^7^ cells/mL. 75 μL aliquots of the cell suspension was incubated with 25 μL aliquots of PuroA peptide (to a final peptide concentration of 64 μg\mL, 125 μg\mL and 250 μg\mL). The mixtures were incubated for 1 h at 30 °C, the cells collected by centrifugation at 1000 × *g* for 5 min, then washed twice by 0.1 M phosphate buffered saline (PBS, pH 7.4), and resuspended in 100 μL MilliQ water. 30 μL aliquots of the above cell suspensions were spotted onto clean glass slides and air-dried. The slides were fixed in 2.5% glutaraldehyde in PBS (pH 7.4) for 2 h in a humid chamber (consisting of a petri dish containing a filter paper soaked in sterile water), then washed with 0.1 M PBS for 10 min and dehydrated in an ethanol gradient (50%, 60%, 70%, 80%, 90% and 100%). The slides were freeze-dried overnight and coated in a Dynavac CS300 unit with carbon and gold, followed by attachment of double-sided conducting carbon tape to the slide for better conductivity. The samples were analysed using a ZEISS supra 10 VP field emission SEM (Carl Zeiss, NY, USA) and images captured at different magnifications and further analyzed in ImageJ to measure the pore size.

### Minimum inhibitory concentration assay

The MIC was determined by the microtitre broth dilution method. *C. albicans* cultures were adjusted to a 0.5 McFarland turbidity standard (approximately 1–5 × 10^6^ cells/mL), then diluted 1:200 with PDB to a final concentration of 0.5–2 × 10^3^ cells/mL. Peptides in 25 μL volumes were added to sterile, flat-bottomed, polypropylene 96-well microtitre plates (Corning, USA) and a two-fold serial dilution (final concentrations of 250 to 0.5 μg mL-1) performed. The wells were inoculated with 75 μL of yeast suspension and the plates incubated at 30 °C for 24 h. Positive growth controls (no peptide) and sterility controls (PDB with no cells and no antimicrobial agents) were also included. The MIC was defined as the lowest peptide concentration that completely inhibited bacterial growth^44^, determined by measuring the absorbance at 595 nm using a Microplate reader (POLARstar Omega, Germany).

### Time-kill assay

A cell suspension of *C. albicans* (at 0.5–2 × 10^3^ cell/mL) was prepared as described above. A 25 μL aliquot of PuroA and FITC-labelled PuroA peptides (final concentration 1 × MIC) was incubated with 75 μL of cell suspension for 0, 10, 20, 30, 40, 50, 60, 120, 180 and 240 min at 30 °C. After these time periods, a 10 μL aliquot was taken and serially diluted with sterile PBS and plated onto PDA. After overnight incubation at 30 °C, the viable cell numbers were determined. Three independent experiments will be performed. Data were analyzed by ANOVA using IBM SPSS Statistical version 23 for windows software and differences with *P* values <0.05 were considered statistically significant.

### Cell cycle analysis

Log phase *C. albicans* cells (1 × 10^6^ cells) cultured in PDB, were harvested and incubated with 125 μg\mL of PuroA for 25 min only at 30 °C. After 25 min incubation, the cells were centrifuged, washed with sterile PBS to remove any extracellular peptide residues, as it was found that incubation the cells with the peptide for longer than 25 min, most of the cells were already killed. The cells pellet was resuspended in a fresh PDB medium and incubated for 8 h at 30 °C with agitation. After incubation, the cells were collected by centrifugation (2000 rpm) for 5 min at 4 °C, washed with PBS, resuspended and fixed in 70% ice cold ethanol overnight. The fixed cells were then harvested by centrifugation and resuspended in 500 μL PBS containing 200 μg RNase and allowed to react for 2 h at 37 C. Subsequently, for DNA staining, 500 μL of propidium iodide (PI) were added to the suspension to make a final concentration of 50 μg\mL and the samples were incubated for 1 h at 4 C in the dark. Cell cycle analysis was performed using Attune NxT Flow Cytometer (Invitrogen™; USA), and the results were analyzed using Attune NxT software.

## Additional Information

**How to cite this article**: Shagaghi, N. *et al*. Revealing the sequence of interactions of PuroA peptide with *Candida albicans* cells by live-cell imaging. *Sci. Rep.* **7**, 43542; doi: 10.1038/srep43542 (2017).

**Publisher's note:** Springer Nature remains neutral with regard to jurisdictional claims in published maps and institutional affiliations.

## Supplementary Material

Supplementary Information

Supplementary Video 1

Supplementary Video 2
