# Supplementary material for: Revealing the sequence of interactions of PuroA peptide with Candida albicans cells by live-cell imaging
Source: Sci Rep. 2017 Mar 2;7:43542. doi: 10.1038/srep43542 (PMC5333355; doi:10.1038/srep43542)
Supplement: Supplementary Information [file srep43542-s1.pdf]

Supplementary Information

Revealing the sequence of interactions of PuroA peptide with *Candida albicans* cells by live-cell imaging

Nadin Shagaghi[1], Mrinal Bhave[1], Enzo A. Palombo[1], Andrew H. A. Clayton[2, *]

[1] Department of Chemistry and Biotechnology, Faculty of Science, Engineering and Technology, Swinburne University of Technology, PO Box 218, Hawthorn, VIC, Australia

[2] Centre for Micro-Photonics, Faculty of Science, Engineering and Technology, Swinburne University of Technology, PO Box 218, Hawthorn, VIC, Australia

[*] Corresponding author:

Andrew H. A. Clayton

Email: aclayton@swin.edu.au

Phone: +61-3-9214-5719

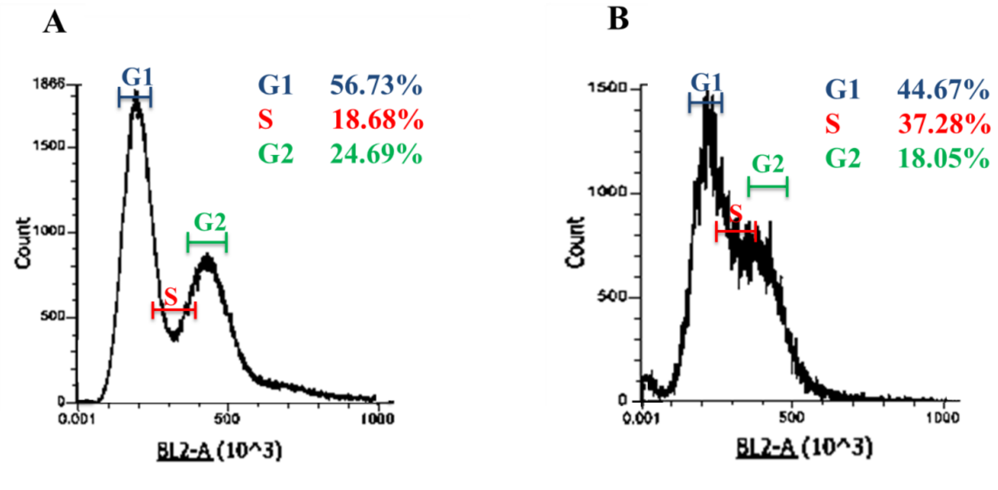

Figure S1. Cell cycle analysis of *C. albicans* cells after treatment with 125 μg\mL of PuroA using flow cytometer. (A) The cell cycle progression of untreated cells. **(B)** The cell cycle progression of treated cells with PuroA for 25 min.

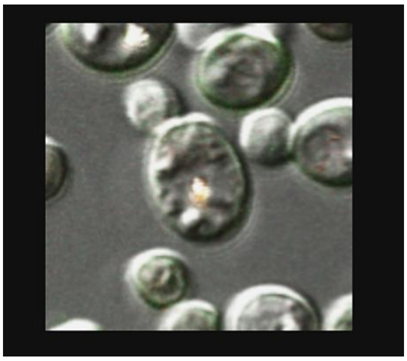

Movie S1. Seventy five minutes of time-lapse imaging of a *C.albicans* cell that is attacking with PuroA, the cell nucleus stained with SYTO 85 Orange before addition of 8 μg/mL FITC-PuroA. To easily localize the peptide, the red channel (SYTO 85), the green channel (FITC-PuroA) and Phase contrast are merged. Injection of FITC-PuroA occurs at t = 0 min.

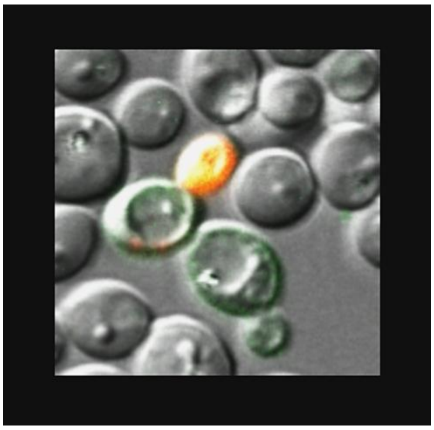

Movie S2. Seventy minutes of time-lapse imaging of a *C.albicans* cell during a typical attack with PuroA, showing when the PI influx occurs. The red channel (PI), the green channel (FITC-PuroA) and Phase contrast are merged. Injection of FITC-PuroA and PI occurs at t = 0 min.
